# Myofunctional Therapy App for Severe Apnea–Hypopnea Sleep Obstructive Syndrome: Pilot Randomized Controlled Trial

**DOI:** 10.2196/23123

**Published:** 2020-11-09

**Authors:** Carlos O'Connor-Reina, Jose Maria Ignacio Garcia, Elisa Rodriguez Ruiz, Maria Del Carmen Morillo Dominguez, Victoria Ignacio Barrios, Peter Baptista Jardin, Juan Carlos Casado Morente, Maria Teresa Garcia Iriarte, Guillermo Plaza

**Affiliations:** 1 Otorhinolaryngology Department Hospital Quironsalud Marbella Marbella Spain; 2 Otorhinolaryngology Department Hospital Quironsalud Campo de Gibraltar Palmones (Cadiz) Spain; 3 Pulmonology Department Hospital Quironsalud Marbella Marbella Spain; 4 Pulmonology Department Hospital Quironsalud Campo de Gibraltar Palmones (Cadiz) Spain; 5 Otorhinolaryngology Department Clinica Universitaria de Navarra Pamplona Spain; 6 Otorhinolaryngology Department Hospital Universitario Virgen de Valme Sevilla Spain; 7 Otorhinolaryngogy Department Hospital Universitario de Fuenlabrada Universidad Rey Juan Carlos Madrid Spain; 8 Otorhinolaryngogy Department Hospital Sanitas la Zarzuela Madrid Spain

**Keywords:** myofunctional therapy, oropharyngeal exercises, mHealth, sleep apnea, smartphone app, app, sleep, therapy, apnea, randomized trial, efficacy

## Abstract

**Background:**

Myofunctional therapy has demonstrated efficacy in treating sleep-disordered breathing. We assessed the clinical use of a new mobile health (mHealth) app that uses a smartphone to teach patients with severe obstructive sleep apnea–hypopnea syndrome (OSAHS) to perform oropharyngeal exercises.

**Objective:**

We conducted a pilot randomized trial to evaluate the effects of the app in patients with severe OSAHS.

**Methods:**

Forty patients with severe OSAHS (apnea–hypoxia index [AHI]>30) were enrolled prospectively and randomized into an intervention group that used the app for 90 sessions or a control group. Anthropometric measures, Epworth Sleepiness Scale (0-24), Pittsburgh Sleep Quality Index (0-21), Iowa Oral Performance Instrument (IOPI) scores, and oxygen desaturation index were measured before and after the intervention.

**Results:**

After the intervention, 28 patients remained. No significant changes were observed in the control group; however, the intervention group showed significant improvements in most metrics. AHI decreased by 53.4% from 44.7 (range 33.8-55.6) to 20.88 (14.02-27.7) events/hour (*P*<.001). The oxygen desaturation index decreased by 46.5% from 36.31 (27.19-43.43) to 19.4 (12.9-25.98) events/hour (*P*=.003). The IOPI maximum tongue score increased from 39.83 (35.32-45.2) to 59.06 (54.74-64.00) kPa (*P*<.001), and the IOPI maximum lip score increased from 27.89 (24.16-32.47) to 44.11 (39.5-48.8) kPa (*P*<.001). The AHI correlated significantly with IOPI tongue and lip improvements (Pearson correlation coefficient −0.56 and −0.46, respectively; both *P*<.001). The Epworth Sleepiness Scale score decreased from 10.33 (8.71-12.24) to 5.37 (3.45-7.28) in the app group (*P*<.001), but the Pittsburgh Sleep Quality Index did not change significantly.

**Conclusions:**

Orofacial exercises performed using an mHealth app reduced OSAHS severity and symptoms, and represent a promising treatment for OSAHS.

**Trial Registration:**

Spanish Registry of Clinical Studies AWGAPN-2019-01, ClinicalTrials.gov NCT04438785; https://clinicaltrials.gov/ct2/show/NCT04438785

## Introduction

### Background

Obstructive sleep apnea–hypopnea syndrome (OSAHS) is a serious health problem worldwide [1], and is associated with morbidities such as hypertension, arrhythmia, and cerebrovascular diseases. The classic treatment of this syndrome is based on dietary measures, weight loss, and exercise, and the use of continuous positive airway pressure (CPAP). Other treatment options include upper airway surgery, mandibular advancement devices, and upper airway stimulation devices (UASDs) that bring the tongue forward to prevent it from falling backward and collapsing the airway. The success rates of treating the airway obstacle or correcting the muscles vary. Indications and the success rate for all treatments depend on patient compliance with the treatment and the severity of the disease [2].

Patients with OSAHS present with impaired sensorimotor deficits located in the upper airway muscles [3]. These deficits are associated with apraxia [4], hypotonia [5], and changes in the type of muscle fibers from type I to types IIa and IIb, which lead to early fatigue and can interfere with the ability to perform prolonged exercise [6]. These deficits can lead to impairment of proprioceptive acuity in the upper airway muscles [4]. The best rehabilitation for improving this pathology is proprioceptive training in association with visual or acoustic feedback [7].

Myofunctional therapy is one of the newest treatments for sleep-disordered breathing [8], which is based on daily exercises using the oropharyngeal muscles in an attempt to strengthen them and to facilitate opening of the airway. OSAHS originates from suboptimal function of the dilator muscles of the airway. Therefore, myofunctional therapy is designed, theoretically, to deal with the underlying mechanism of this disease [9]. The patient is instructed to perform these exercises regularly for 20-40 minutes daily for at least 3 months [10]. In some cases, patients perform the exercises independently at home without substantial feedback and without giving precise information to the therapist about their performance of the exercises, and this type of therapy is associated with low adherence [11].

Most existing mobile health (mHealth) apps for OSAHS focus on the diagnosis of snoring or OSAHS [12], and a few are designed to promote adherence to treatment with CPAP [13]. None of these apps is used alone to treat OSAHS. Mobile technology may be valuable for treating people with OSAHS because it may promote patient empowerment and self-management [12].

Therefore, we conducted this pilot randomized trial to evaluate a new mHealth app based on proprioceptive training, which was designed to promote oropharyngeal exercises through interactions with a smartphone. In this prospective, randomized, multicenter clinical study, we evaluated adherence to the app and its effectiveness in a group of patients with severe OSAHS, as identified by an apnea–hypoxia index (AHI)>30 compared with a control group of similar patients who did not participate in the intervention.

### Objectives

#### Primary Objectives

The main objectives were to study the effects of the AirwayGym app on adherence to myofunctional therapy and on the AHI in patients recently diagnosed with severe OSAHS (AHI>30).

#### Secondary Objectives

The secondary objectives were to evaluate the change in the oxygen desaturation index (ODI), use of the Iowa Oral Performance Instrument (IOPI) score to evaluate the effects of the app on the tone of the genioglossus and buccinator muscles, and use of the Epworth Sleepiness Scale and Pittsburgh Sleep Quality Index questionnaires to evaluate subjective morning somnolence and sleep quality.

## Methods

### Study Overview

This was a nonsponsored study coordinated by the Pulmonology and Otolaryngology Departments of Quirónsalud Marbella Hospital and Campo de Gibraltar Hospital, Andalucia, Spain. The protocol was designed and written by the authors and is available in Multimedia Appendix 1. The protocol was approved by the governmental review board (AWGAPN-2019-01). All patients provided informed consent. An independent data and safety monitoring board regularly reviewed data on serious or nonserious adverse events and study quality. The authors vouch for the accuracy and completeness of the data reported and for the fidelity of the study to the protocol. All investigators were GCP-certified.

The CONSORT (Consolidated Standards of Reporting Trials) checklist [14] is shown in Multimedia Appendix 2.

### Design

This was a prospective controlled quasiexperimental clinical study in patients with severe OSAHS (AHI>30).

### Participants and Recruitment

Patients newly diagnosed with severe OSAHS based on the results of polysomnography or respiratory polygraphy with measures of AHI and oxygen saturation were recruited offline in a clinical setting. All sleep studies were interpreted manually by a sleep technician according to the standard criteria of the American Academy of Sleep Medicine Manual for the Scoring of Sleep and Associated Events [15], and the interpretations were reviewed by certified physicians.

Information about the inclusion and exclusion criteria, evaluation of the type of smartphone used, previous experience with the app, and the study protocol are provided in Multimedia Appendix 1.

All patients agreed to participate and provided offline informed consent. At the initial visit, participants were evaluated by an otorhinolaryngologist who performed rhinofibrolaryngoscopy, Friedman staging, the Marchesani protocol [16], and examination of temporomandibular joint dysfunction. Patients with grade IV tonsils, complete nasal obstruction, ankyloglossia, or problems with temporomandibular joint dysfunction were excluded from the study. In a second visit, anthropometric variables, including weight, height, and neck and waist circumferences, were measured, and the BMI was calculated. The Friedman staging, Epworth Sleepiness Scale, and Pittsburgh Sleep Quality Index questionnaires were completed, and IOPI lingual and buccinator scores were obtained.

### Randomization

Randomization was based on the consecutive order of patient enrollment. A pulmonologist specialist allocated odd-numbered patients to the AirwayGym app group and even-numbered patients to the control group.

### Intervention

Participants in the AirwayGym group were instructed about the use of the app and the exercises to perform for 20 minutes daily. Follow-up visits for both the AirwayGym and control groups occurred after 1 month (visit 3) and 3 months (visit 4). At these visits, all variables were measured again and the questionnaires were completed, and the patients were asked whether they were using any other therapies. In the final visit at 3 months, polysomnography or polygraphy was performed for both groups. The total study duration for each participant was 3 months.

Participants were excluded from the study if they were lost to follow up because they did not attend hospital visits or if they lost ≥5% of body weight during the study. Patients in the AirwayGym group were also excluded if they did not perform ≤85% of the scheduled exercise sessions, as monitored by the app.

### Myofunctional Therapy App

Participants in the AirwayGym group committed to using the AirwayGym app. This app was created as a collaboration between the sleep units of Quirónsalud Marbella Hospital and Campo de Gibraltar Hospital. The app can be thought of as a portable fitness app except that the user is intended for patients rather than athletes, and therapists rather than trainers provide the instructions. The novelty of this app is that it is the first app in the health care market that allows the patient to interact directly with the smartphone without needing any other device. The app focuses on sleep apnea disease and improving proprioceptive deficits. When used with the app, the phone provides acoustic feedback about the efficacy of the exercises performed.

**Figure 1:**
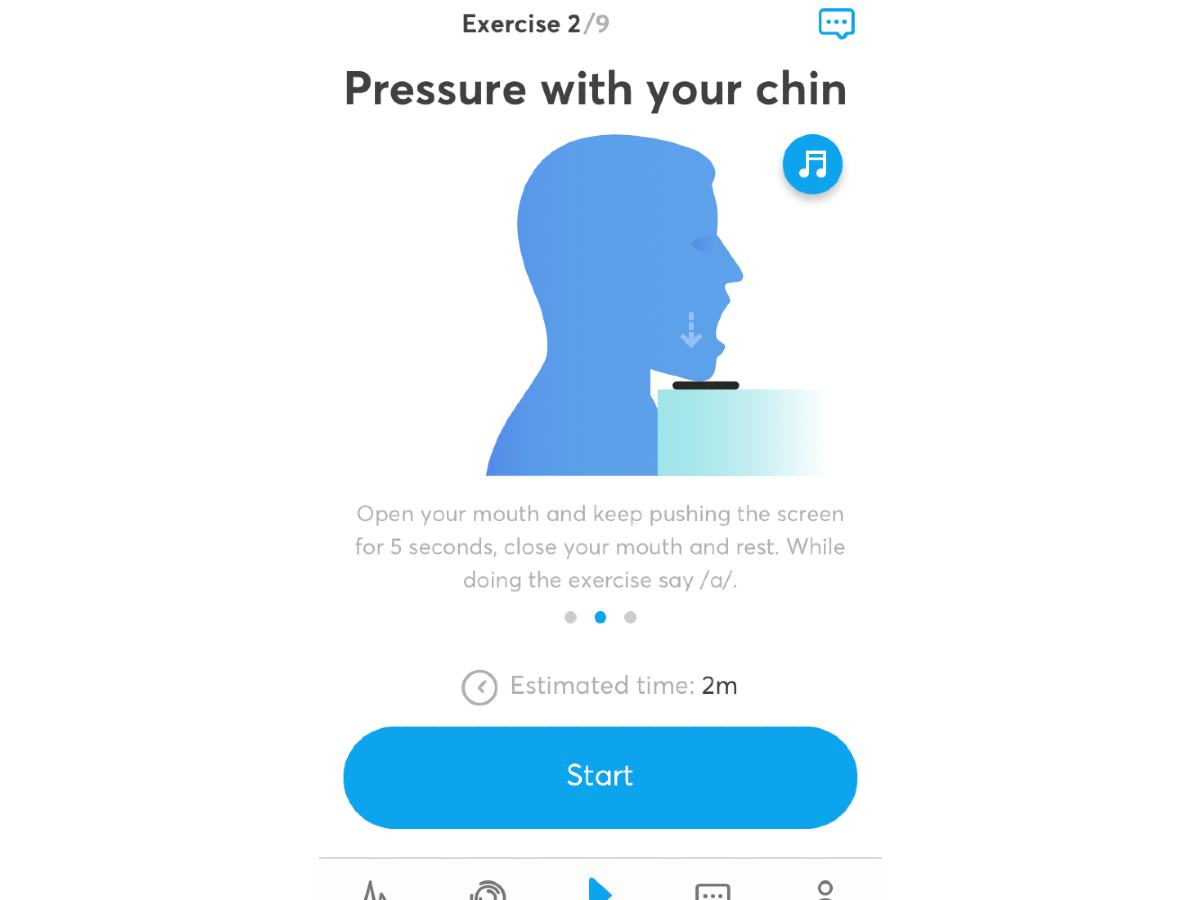
Screenshot of a gif showing an exercise.

**Figure 2:**
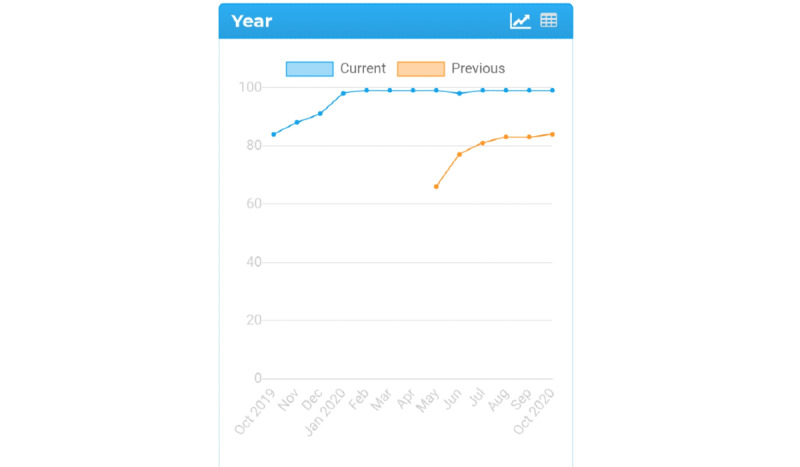
Screenshot of a patient’s progress in following the exercises for 1 year.

The app includes 9 exercises based on myofunctional therapy (Figure 1) that are aimed at improving the tonicity of the various muscles involved in the pathogenesis of OSAHS. Before every exercise, an animated demonstration and a video show the patient how to perform the exercise. After each exercise, the patient receives visual, acoustic, and tactile feedback about the success of their performance as a point score. When the patient finishes the exercises, the results are saved on a networked online storage (in the cloud), and a therapist can evaluate the patient’s performance of the exercises. Users of the app can follow the progress of their daily activity over time (Figure 2). A chat function is available through which the patient can contact the therapist directly. Additional information can be found on the AirwayGym webpage [17]. This app complies with regulations 2002/58/CE and (UE) 2016/679 concerning data protection. The app was provided free to each participant.

The main objective of the exercises in the app is to increase the tone of the extrinsic muscles of the tongue (genioglossus, hyoglossus, styloglossus, and palatoglossus). The exercises are based on those described elsewhere [10] and have been adapted to allow feedback using a smartphone (see Multimedia Appendices 3-12).

### Sleep Laboratory Procedures

Standard laboratory polysomnography (Somté PSG, Compumedics Ltd 2006, Abbotsford, Australia) was performed according to the technical specifications of the American Academy of Sleep Medicine [15]. The recorded variables were obtained using electroencephalography (C3-A2, C4-A1, O1-A2, O2-A1), electrooculography (2 channels), chin and leg electromyography, and electrocardiography. Frontal electrodes were not used. Respiratory variables were measured using linearized nasal pressure prongs and the flow waveform of the oronasal thermal signals. Respiratory effort signals were measured through inductive bands that recorded ribcage and abdominal movements. Oxygen saturation, body position, and snoring were also registered.

Respiratory polygraphy was performed using an Embletta portable diagnostic system (ResMed, Sydney, Australia) according to the technical specifications of the American Academy of Sleep Medicine [15]. Measurements were obtained using a snoring sensor, nasal thermistor, and nasal pressure cannula to register airflow; thoracic and abdominal belts to assess ribcage and abdominal movements; electrocardiography; actigraphy to detect body position; oxygen saturation; and heart rate.

All patients were evaluated using the same testing procedure (polysomnography or respiratory polygraphy) before and after the intervention. The results for each participant were analyzed manually by a technician who was blinded to the participant’s assigned group.

The pulmonologist’s medical evaluation was used to determine which test was chosen for each patient. Apnea and hypopnea were analyzed and scored according to the following criteria. Hypopnea was defined as a ≥30% decrease in airflow signal amplitude lasting ≥10 seconds and accompanied by ≥3% oxygen desaturation. Apnea was defined as a ≥90% decrease in airflow signal amplitude lasting ≥10 seconds. The ODI was used to quantify oxygen desaturation ≥3% Both tests were used to define moderate OSAHS as an AHI of 15-29.9 events/hour of sleep and severe OSA as ≥30 events/hour of sleep.

### IOPI Measurements

Detailed information about this device and measurements is provided in Multimedia Appendix 1.

### Sample Size Derivation

The effectiveness of use of the app for performing myofunctional therapy in patients with severe OSAHS was evaluated using the percentage changes in the AHI observed during follow up as the primary outcome measure. This percentage was calculated from results reported in previous studies of myofunctional therapy [10,18,19]. Based on an α level of .05 and power of 0.80, we estimated that 30 participants were required. To account for potential loss during the inclusion process (including patients with selection bias), early withdrawal, or loss to follow up, we doubled the sample size to 60. The sample size was calculated using XLSTAT (v16 Addinsoft France).

### Data Analysis

Data were collected in a database. Nominal variables are described by their frequency distribution. Quantitative variables were assessed by calculating the median and IQR. Baseline characteristics of the 2 groups of patients with OSAHS were compared using two-tailed paired *t*-tests for continuous variables and the chi-squared or Fisher exact test for nominal variables. For variables with a skewed distribution, the Mann-Whitney *U* test was used. Pearson correlational analysis was used to assess the associations between the changes in the AHI and changes in possible explanatory variables, including BMI and neck and waist circumferences. A *P* value <.05 was considered to be significant.

## Results

### Patients

**Figure 3:**
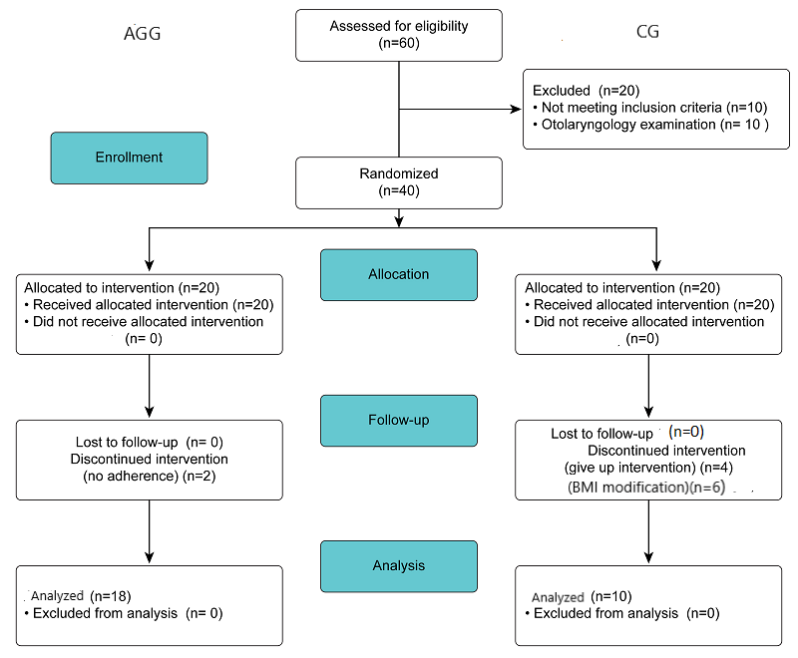
CONSORT flowchart for pilot randomized trials for recruitment of participants in this study. AGG: AirwayGym group; CG: control group.

Of the 60 patients initially recruited, 40 patients were enrolled and randomized from February 2019 to July 2020. Twenty of the 60 patients were excluded, 10 (17%) because of the exclusion criteria and 10 (17%) because of findings in the otorhinolaryngologist’s examination. Six of the 40 patients were excluded because of a change in body weight, 4 voluntarily abandoned the study in the control group, and 2 patients were lost because of poor adherence to therapy in the AirwayGym group. Finally, the data for 28 patients (22 men) were included in the study (Figure 3). Half of the participants in the control group were lost to follow up.

The baseline demographic and sleep characteristics are presented for the two groups in Table 1. There were no significant differences in any characteristics, including age, weight, and BMI, between the two groups. In addition, at the baseline, the AHI did not differ within or between the groups (Table 1).

**Table 1:** Baseline characteristics of the study participants.

Characteristic		Control (n=10)	AirwayGym (n=18)	*P* value
**Anthropometric data**				
	Age (years), median (IQR)	63.9 (56.4-71.38)	59.17 (53.7-64.6)	.19
	Female, n (%)	2 (20)	4 (22)	.95
	Body weight (kg), median (IQR)	87.7 (77.9-97.4)	86.11 (77.4-94.8)	.86
	Height (cm), median (IQR)	170.2 (166.2-174.1)	177.1 (166.3-177.9)	.52
	Waist circumference (cm), median (IQR)	109.1 (97.5-120.6)	109.2 (102.9-115.4)	.98
	Neck circumference (cm), median (IQR)	43.7 (40.3-47)	43.74 (41.03-46.4)	.99
	BMI (kg/m^2^), median (IQR)	29.6 (27.1-32.08)	28.9 (26.8-31.02)	.67
	Friedman stage, n (stage)	2 (I), 3 (II), 2 (III), 3 (IV)	4 (I), 4 (II), 3 (III), 7 (IV)	.95
**Polysomnography data**				
	AHI^a^/hour, median (IQR)	47.36 (38.59-56.13)	44.77 (33.841-55.69)	.70
	ODI^b^, median (IQR)	40.6 (29.46-51.81)	36.31 (27.1-43.43)	.54
**Tone measures (kPa)**				
	IOPI^c^ max tongue^d^, median (IQR)	42 (32.67-51.33)	40.26 (35.32-45.2)	.70
	IOPI max lips^e^, median (IQR)	28.10 (23.7-32.5)	28.3 (24.16-32.47)	.94
**Questionnaires**				
	Pittsburgh Sleep Quality Index, median (IQR)	8.80 (7.12-10.48)	10.1 (8.22-11.99)	.34
	Epworth Sleepiness Scale, median (IQR)	9.3 (6.6-12)	10.47 (8.71-12.24)	.42

^a^AHI: apnea–hypopnea index.

^b^ODI: oxygen desaturation index.

^c^IOPI: Iowa Oral Performance Instrument.

^d^maximum tongue elevation strength.

^e^maximum lip strength.

### Intervention

After the intervention period, none of the variables changed significantly in the control group (Table 2). Conversely, significant changes from before to after the intervention were observed in the AirwayGym group. The adherence to the therapy in the AirwayGym group was 90%. No adverse reactions were registered.

**Table 2:** Changes in variables from the baseline to 3-month follow up in the control and AirwayGym groups.

Variable		Control group (n=10)			AirwayGym group (n=18)		
		Baseline, median (IQR)	After 3 months, median (IQR)	*P* value	Baseline, median (IQR)	After 3 months, median (IQR)	*P* value
**Anthropometric data**							
	Body weight (kg)	87.7 (77.9-97.4)	87.3 (78.03-95.66)	.95	86.1 (77.4-94.8)	86.00 (77.93-94.06)	.92
	Height (cm)	170.2 (166.2-174.1)	169.80 (166.27-174.12)	.98	177.1 (166.3-177.9)	172.6 (167.3-177.9)	1.00
	Waist circumference (cm)	109.1 (97.5-120.6)	108.5 (96.9-120.10)	.94	109.2 (102.9-115.4)	108.84 (102.7-114.9	.92
	Neck circumference (cm)	43.7 (40.3-47)	43.5 (40.33-46.67)	.92	43.74 (41.03-46.4)	44.6 (42.12-47.2)	.60
	BMI (kg/m^2^)	29.63 (27.1-32.08)	29.6 (27.35-31.84)	.94	28.9 (26.8-31.02)	28.81 (26.79-30.83)	.92
**Polysomnography data**							
	AHI^a^ (events/hour)	47.36 (38.59-56.13)	35.00 (31.2-38.7)	.07	44.77 (33.84-55.69)	20.88 (14.02-27.74)	<.001
	ODI^b^	40.64 (29.46-51.81)	32.03 (24.14-39.91)	.17	36.31 (27.19-43.43)	19.4 (12.9-25.98)	.003
**Tone measures (kPa)**							
	IOPI^c^ max tongue^d^	42 (32.67-51.33)	44.2 (34.1-54.2)	.72	39.83 (35.32-45.2)	59.06 (54.74-64.00)	<.001
	IOPI max lips^e^	28.10 (23.7-32.5)	31.3 (26.6-35.9)	.27	27.89 (24.16-32.47)	44.11 (39.5-48.8)	<.001
**Questionnaires**							
	Pittsburgh Sleep Quality Index	8.80 (7.12-10.48)	9.78 (7.2-12.11)	.50	10.2 (8.22-11.99)	8.28 (5.97-10.35)	.22
	Epworth Sleepiness Scale	9.3 (6.6-12.0)	9.6 (6.8-12.3)	.86	10.33 (8.71-12.24)	5.37 (3.45-7.28)	<.001

^a^AHI: apnea–hypopnea index.

^b^ODI: oxygen desaturation index.

^c^IOPI: Iowa Oral Performance Instrument.

^d^maximum tongue elevation strength.

^e^maximum lip strength.

### Anthropometric Data

The anthropometric measures did not change significantly in the intervention group (Table 2).

### Laboratory and IOPI Measurements

**Figure 4:**
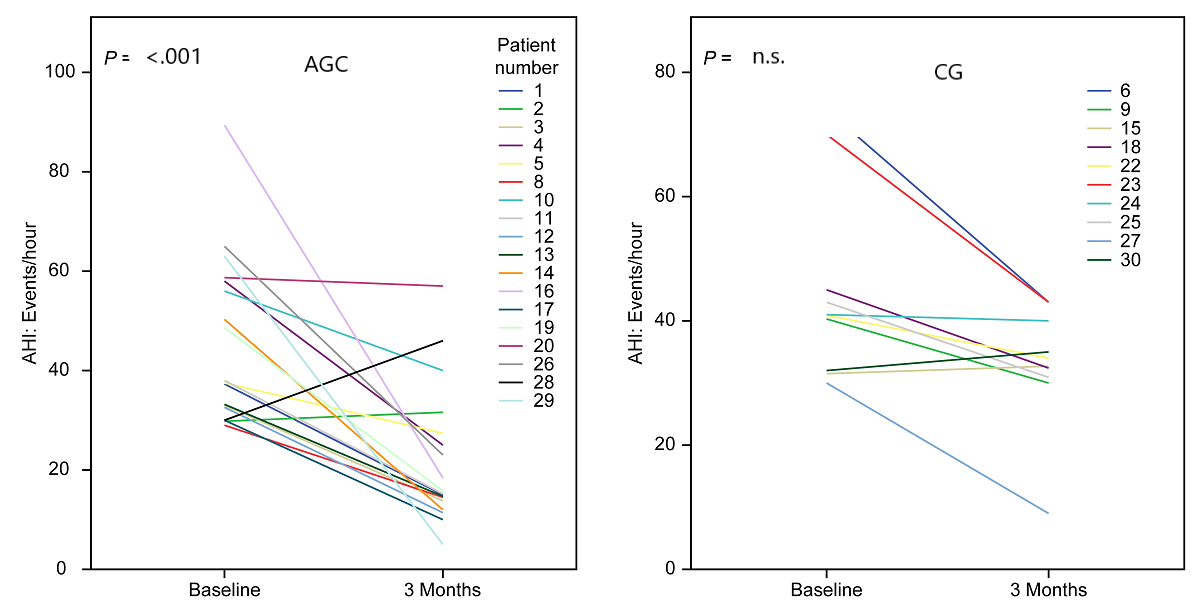
Apnea–hypopnea index (AHI) before (baseline) and after 3 months in patients with severe obstructive sleep apnea. Intragroup comparison from before to after the study was performed using the Wilcoxon test owing to the skewed data distribution. CG: control group; AGG: AirwayGym group.

**Figure 5:**
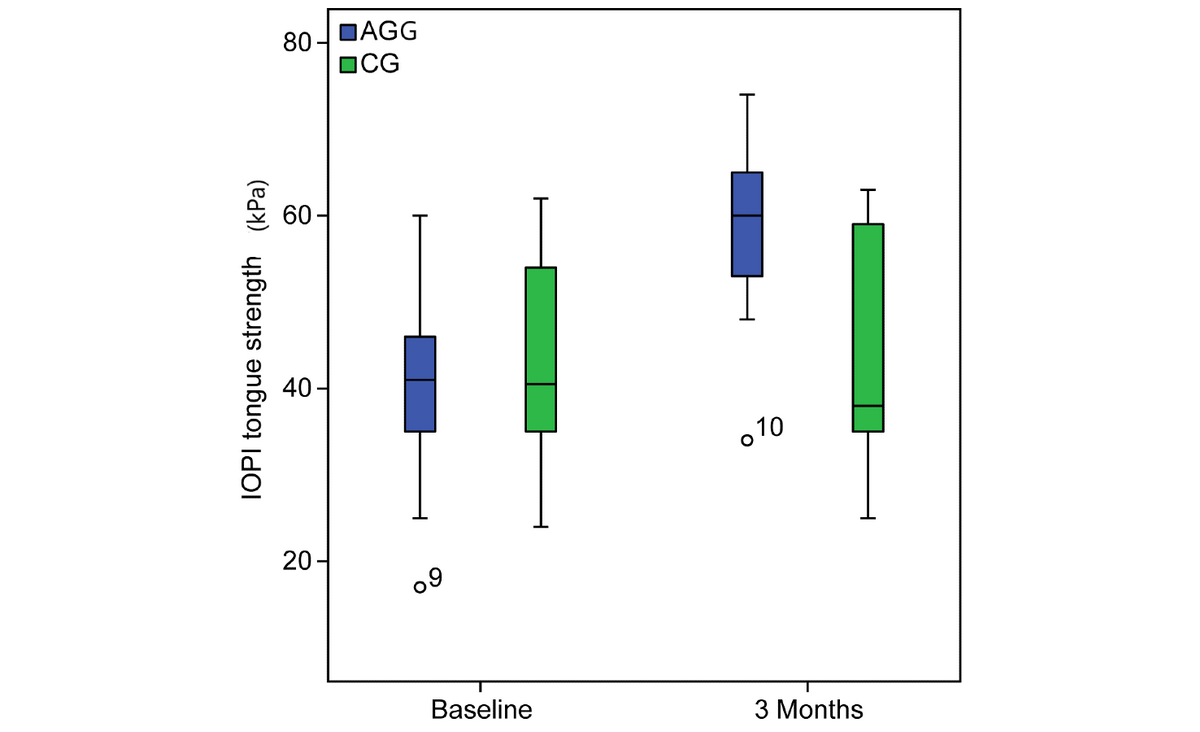
Iowa Oral Performance Instrument (IOPI) tongue strength (kPa) at baseline and after 3 months in patients with severe obstructive sleep apnea. The intragroup comparison is shown from before to after the study. AGG: AirwayGym group; CG: control group.

**Figure 6:**
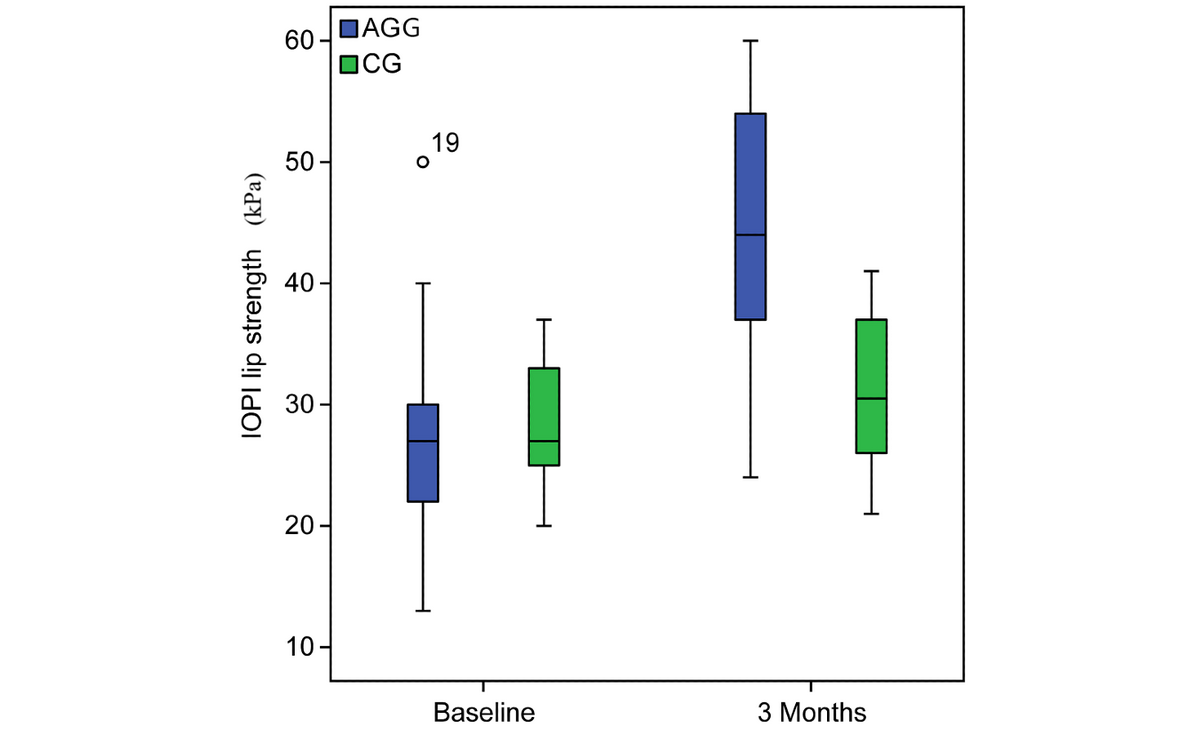
Iowa Oral Performance Instrument (IOPI) lip strength (kPa) at baseline and after 3 months in patients with severe obstructive sleep apnea. The intragroup comparison is shown from before to after the study. AGG: AirwayGym group; CG: control group.

The AHI decreased by 53.36% from 44.7 (IQR 33.8-55.6) to 20.88 (IQR 14.02-27.7) events/hour (*P*<.001) (Figure 4). The ODI score decreased by 46.5% from 36.31 (IQR 27.19-43.43) to 19.4 (IQR 12.9-25.98) events/hour (*P*=.003). The IOPI maximum tongue elevation strength score increased from 39.83 (IQR 35.32-45.20) to 59.06 (IQR 54.74-64.00) kPa (*P*<.001) (Figure 5). The IOPI maximum lip score increased from 27.89 (IQR 24.16-32.47) to 44.11(IQR 39.5-48.80) kPa (*P*<.001) (Figure 6). The AHI correlated significantly with improvements in the IOPI tongue and lip scores (Pearson coefficients −0.56, *P*<.001 and −0.46, *P*<.001, respectively).

### Questionnaires

The Epworth Sleepiness Scale score decreased from 10.33 (IQR 8.71-12.24) to 5.37 (IQR 3.45-7.28) (*P*<.001). However, the Pittsburgh Sleep Quality Index did not change significantly in the AirwayGym group.

## Discussion

### Principal Findings

This prospective study showed that an mHealth app that includes education about myofunctional therapy exercises for patients with severe OSAHS helped to improve the AHI and upper airway muscle tone. This is the first report in the literature to show significant correlations between the increase in tone, as measured by the IOPI, and improvements in the AHI in patients with severe OSAHS.

The main criticisms of previous reports on the use of myofunctional therapy to treat OSAHS patients are that the myofunctional therapy included different kinds of exercises and the authors did not explain the reason for improvement [8]. The clinical trial by Guimaraes et al [10] reported a significant reduction in neck circumference, which may explain the effectiveness of the exercises. Our app provides one unique set of exercises with acoustic, visual, and tactile feedback. We found no significant change in neck circumference, which suggests that increasing muscle tone, as measured by the IOPI, helped to improve the AHI.

Eckert [20] reported the use of myofunctional therapy and UASDs as an elective treatment for patients with a hypotonic phenotype associated with OSAHS. Our results may be similar to those obtained with a stimulator [21], but the app costs less and does not appear to be associated with adverse effects. Another difference after myofunctional therapy is that the IOPI scores increased after use of the app, whereas UASDs have not been found to increase IOPI scores [22]. Active voluntary movement is required to increase the muscle tone, but UASDs do not elicit such movement. However, the app must be evaluated for a longer time, 1 year or more, as performed with UASDs, to make definitive conclusions [21].

We believe that the reason for the success of this app in patients with severe OSAHS is that myofunctional therapy must be based on proprioceptive training because of the sensorimotor deficit in the upper airway muscles in these patients [4]. All clinical studies reported to date are based on isometric and isotonic exercises in patients with moderate or severe sleep apnea, and used videos and diagrams [10,13,19]. Our app is based on proprioceptive training using isometric and isotonic exercises, which led to satisfactory results in these patients with severe OSAHS.

The main disadvantages of myofunctional therapy are the poor adherence to therapy [23] and the absence of objective feedback [10,11]. Adherence to myofunctional therapy was high in our study, possibly because of the ease of contact with a practitioner and the acoustic and visual feedback about the patient’s performance given by the app for every exercise [24]. Monthly visits for measurement of IOPI scores can also provide an objective way to evaluate patient progress and to promote adherence.

Instead of using placebo exercises, as employed in other studies [25], we chose to withhold therapy for the control group because there is no separate app that could be used as a control. This study also describes a new method for delivering upper airway exercise training for which there is no comparable study available to date.

The selection of patients suitable for myofunctional therapy is important [18]. The efficacy of this therapy may be limited in patients with restricted tongue movement, permanently blocked nose, or temporomandibular joint disorder. Therefore, patients with OSAHS should be examined by a therapist with knowledge of the use of myofunctional therapy before beginning such therapy [26]. In our study, 10 patients (16.6%) were rejected because of one of these conditions. This app is suitable only for improving upper airway muscle tone and does not correct other myofunctional therapy disorders [27]. Future studies are needed to identify the hypotonic phenotype(s) suitable for this therapy. As we have suggested previously [28], patients with a low IOPI score are good candidates for myofunctional therapy.

Although further evidence of the efficacy of this app is needed, we consider that this therapy will help to improve adherence to other treatments, as has been suggested previously [28-30]. In addition, conventional treatments such as CPAP or sleep surgery have been restricted during the COVID-19 pandemic [31], and this app may be a reasonable therapeutic option, as we have reported recently [24].

Despite the strengths of this pilot study mentioned above, we note several limitations. First, the number of participants was small. Second, we found a significant loss of participants in the control group (50%), who were instructed not to perform any therapy once they had been diagnosed with severe OSAHS. Despite this loss, our sample size was similar to that included in other clinical studies of this therapy [10,18,19]. The number of patients in the control group was low because we found significant differences in the therapy group early during the study. We therefore decided not to enroll more patients in the control group because of the difficulties experienced by patients with severe OSAHS not given appropriate therapy and the restrictions on sleep studies during the COVID-19 pandemic. Third, as this was a nonsponsored pilot trial, we initially attempted to perform polysomnography studies in all patients, but this test slowed our trial. We decided to use laboratory polygraphy because we found no significant differences in the accuracy of the outcomes AHI and ODI between polysomnography and polygraphy provided that the pre- and postintervention measures were obtained by the same team and equipment. Finally, this pilot trial was registered at clinicaltrials.gov during the performance of the study.

### Conclusions

In patients with OSAHS who performed myofunctional therapy exercises using this app, the severity of symptoms decreased and the tone of the upper airway muscles increased after 3 months. This app may represent a promising treatment for OSAHS given its convenience and availability of the mobile phone market.

## Appendix

Multimedia Appendix 1 Study protocol.

Multimedia Appendix 2 CONSORT checklist.

Multimedia Appendix 3 Screenshots from the app.

Multimedia Appendix 4 Video of exercise 1: chromatic snake.

Multimedia Appendix 5 Video of exercise 2: Snake.

Multimedia Appendix 6 Video of exercise 3: Chameleon up.

Multimedia Appendix 7 Video of exercise 4: Chameleon down.

Multimedia Appendix 8 Video of exercise 5: Tongue left cheek.

Multimedia Appendix 9 Video of exercise 6: Tongue right cheek.

Multimedia Appendix 10 Video of exercise 7: Pressure under the chin.

Multimedia Appendix 11 Video of exercise 8: Left mandibular pressure.

Multimedia Appendix 12 Video of exercise 9: Right mandibular pressure.

## Abbreviations

AHI: apnea–hypoxia index
CONSORT: Consolidated Standards of Reporting Trials
CPAP: continuous positive airway pressure
IOPI: Iowa Oral Performance Instrument
mHealth: mobile health
ODI: oxygen desaturation index
OSAHS: obstructive sleep apnea–hypopnea syndrome
UASD: upper airway stimulation device
