# Supplementary material for: Myofunctional Therapy App for Severe Apnea–Hypopnea Sleep Obstructive Syndrome: Pilot Randomized Controlled Trial
Source: JMIR Mhealth Uhealth. 2020 Nov 9;8(11):e23123. doi: 10.2196/23123 (PMC7683258; doi:10.2196/23123)
Supplement: Multimedia Appendix 1 [file mhealth_v8i11e23123_app1.docx]

# Myofunctional Therapy App for Severe Apnea–Hypopnea Sleep Obstructive Syndrome: Pilot Randomized Trial

Spanish Gov: AWGAPN-2019-01

ClinicalTrials.gov: NCT04438785

## Corresponding Author

Carlos O’Connor Reina MD PhD;

Hospital Quiron Salud Marbella, Cohead Department Otorhinolaryngology; Hospital Quiron Salud Campo de Gibraltar, Cohead Department of Otorhinolaryngology.

Phone 0034952780540

coconnor@us.es

## Introduction

Obstructive sleep apnea–hypopnea syndrome (OSAHS) has become one of the most serious health problems worldwide [1]. Because of its association with obesity, the prevalence and incidence of OSAHS have been increasing, and the condition is associated with morbidities such as cardiovascular and cerebrovascular diseases. The classic treatment of this disease is based on dietary measures, weight loss, exercise, and use of a continuous positive airway pressure (CPAP) machine, a device that reduces the collapsibility of the upper airway by emitting a flow of air. Other options are upper airway surgery to treat obstacles in the airway or to correct the muscles that do not perform their function properly, and mandibular advancement devices (MADs), which protrude the tongue forward to prevent it falling backward and collapsing the airway. The success rates for such procedures are variable, and the indications and level of success depend on the severity of the disease and patient adherence to the treatment [1].

Myofunctional therapy (MT) is one of the newest treatments for sleep-disordered breathing [2]. MT is based on daily exercises using the oropharyngeal muscles that aim to strengthen the muscles and to facilitate opening of the airway. OSAHS originates from the lack of optimal function of the dilator muscles of the airway, and MT is designed, theoretically, to correct the underlying mechanisms causing this disease [3]. MT includes oropharyngeal exercises, which are described using diagrams, videos, and instructions provided by a myofunctional therapist weekly. The patient is instructed to perform the exercises regularly for 20-40 min daily for at least 3 months. In some cases, patients perform the exercises by themselves at home without substantial feedback and without providing precise information to the therapist about their performance of the exercises [4].

Most existing mobile health (mHealth) apps for patients with OSAHS focus on the diagnosis of snoring or OSAHS [5], although a few are designed to promote adherence to treatment with CPAP [6]. To our knowledge, none of these apps focus on the treatment of OSAHS. However, mobile technology may be valuable in treating OSAHS patients because of its potential to promote patient empowerment and self-management [7].

Exercise and weight loss are two of the best treatments for OSAHS [7,8]. Given time limitations, we consider that patients are more likely to perform exercises if they are able to do so while sitting comfortably and watching television. Therefore, we designed and developed a novel mHealth app to teach and promote the use of oropharyngeal exercises using a smartphone. In this prospective, randomized, multicenter clinical trial, we will assess adherence to the use of the app and its effectiveness in a group of patients with severe OSAHS compared with a control group.

### Hypothesis

Regular use of the AirwayGym app, which is designed to help patients perform MT, will increase adherence to MT and improve symptoms in patients with severe disease OSAHS (apnea–hypoxia index (AHI) >30) by increasing the tone of the upper airway muscles.

## Objectives

### Primary Objective

The main objective is to study the evolution of the efficacy of the AirwayGym app for performing MT in patients diagnosed with severe OSAHS.

### Secondary Objectives

The secondary objectives are as follows.

1. To evaluate the effects of MT used via the app on the AHI in patients diagnosed with severe OSAHS.

2. To evaluate the effects of the app on the O_2_ desaturation index in these patients.

3. To evaluate the effects of the app on the tone of the genioglossus and buccinator muscles as assessed using the Iowa Oral Performance Instrument (IOPI).

4. To evaluate subjective ratings of morning somnolence and sleep quality as assessed using the Epworth Sleepiness Score and Pittsburgh Sleep Quality Index.

## Methods

### Design

This will be a prospective, controlled, quasi-experimental pilot clinical trial in patients with severe OSAHS (AHI > 30).

### Place

Patients recently diagnosed with severe OSAHS in the pneumology units of Hospital Quirónsalud Marbella and Hospital Campo de Gibraltar will be recruited.

### Population

Patients recently diagnosed with severe sleep apnea and accepted to participate in this pilot clinical trial.

### Inclusion Criteria

- age 18-75 years
- recently diagnosed with severe sleep apnea but with no previous experience with this pathology
- provision of informed written consent.

### Exclusion Criteria

- BMI > 40 kg/m^2^
- inability to complete the questionnaires
- severe drug or alcohol abuse
- use of hypnotic medication
- uncontrolled coronary disease
- decompensated heart failure
- history of stroke
- systemic disease associated with an inflammatory-related entity (e.g., arthritis, sarcoidosis, vasculitis, lupus)
- neuromuscular disease (e.g., Duchenne muscular dystrophy)
- craniofacial deformity
- active oncology
- any previous use of MT treatment or other treatments for sleep apnea that could affect the study results (e.g., surgery, MAD, or CPAP).

After being accepted into the study and signing the informed consent form, the patient will be examined by an ear, nose, and throat (ENT) specialist to rule out the following conditions:

- severe upper airway obstruction (complete nose obstruction, tonsils grade III/IV),
- presence of tongue-tie (Marchesani protocol) (9) that limits tongue movements,
- antecedents or presence of temporomandibular joint disorder.

Patients with any of these features will be excluded from the study even after initial acceptance.

### Sample Size Calculation and Sampling Procedure

Evaluation of the effectiveness of the app for MT in patients with severe OSAHS will be based on the percentage changes in the AHI observed during the follow-up in the longitudinal study. This percentage has been calculated using results reported in previous MT studies [10]. Assuming a statistical confidence level of 95%, power of 80%, and estimated improvement of the AHI of 60%, we have calculated that a sample size of 30 patients will be needed (15 in the intervention group, 15 in the control group). To compensate for the potential loss during the inclusion process (including patients with selection bias), early withdrawal, or loss during follow-up, we will double the sample size to 60 (30 in each group). The sample size was calculated using XLSTAT (v16, Addinsoft France).

Patients will be randomized in consecutive order of enrollment, with odd-numbered patients allocated to the intervention group and even-numbered patients to the control group. Patients without a smartphone will be assigned directly to the control group.

## Parameters of Interest

Age, sex, weight, height, BMI, waist circumference (at the umbilicus), neck circumference, IOPI tongue and lip cores, AHI, O_2_ desaturation index, and Friedman tongue stage will be assessed. Patients will complete the Epworth Sleepiness Score and Pittsburgh Sleep Quality Index questionnaires.

## Development and Use of the App

The intervention group will use the mHealth AirwayGym app.

This app is a smartphone app created in a collaboration between the sleep units of Hospital Quirónsalud Marbella and Hospital Campo de Gibraltar and their engineering informatics departments and developed by Apnea Bye. The app was developed in November 2018.

We think of this app as a portable fitness app, except that patients and not athletes use the app, and therapists instead of trainers provide the instruction. The novelty of this app is that it is the first in the health-care market in which the patient can interact directly with the smartphone without any other device. The app focuses on sleep apnea disease and improving proprioceptive deficits. The app includes 9 exercises based on MT aimed at improving the tonicity of the various muscles involved in the pathogenesis of OSAHS. Before every exercise, an animated demonstration and a video show the patient how to perform the exercise . The user can follow the progress of their activity daily over time . After each exercise, the patient receives feedback about the success of their performance as a point score . When the patient finishes the exercises, the results are saved on a networked online storage (in the cloud), and a therapist can evaluate the patient’s performance of the exercises . A chat function is available through which the patient can contact the therapist directly. Therapists can also use the app to enroll and follow up their patients. See screen shots from multimedia appendix 2.

The app sends a reminder every 5 days if the patient “forgets” to perform the exercises. An English-language version of this app is now available; the Android app is available via Google Play and the iOS version via the App Store. Full information about the app is provided at https://airwaygym.App/.

This app was developed using the most cutting-edge technologies (e.g., Ionic, Angular provided by Google and TypeScript by Microsoft) and the most consolidated software languages (e.g., HTML5, CSS3, and PHP). The app takes advantage of 3D Touch technology, which accurately measures the pressure applied to the mobile screen and is available in the latest Apple devices. This app complies with regulations 2002/58/CE and (UE) 2016/679 about data protection.

## Exercises Using the App

The main objective of the exercises included in the app is to increase the tone of the extrinsic muscles of the tongue (genioglossus, hyoglossus, styloglossus, and palatoglossus). The exercises are based on those described by Guimaraes in 2009 (8) and have been adapted to allow the patient to obtain feedback using the app. For reasons related to hygiene, we recommend that users cover the screen with cling film or a hypoallergenic plastic wrap when performing the exercises. See video description and multimedia appendix 3 to 11.

Patients in the control group will not be given any therapy for OSAHS for 3 months.

All patients will visit their hospital once a month for measurement of variables (weight, height, BMI, and neck and waist circumferences), and to complete the questionnaires.

## Hospital Visits

### Selection Visit

Patients newly diagnosed with severe OSAHS based on the results of laboratory polysomnography or polygraphy with measures of AHI and O_2_ desaturation index will be recruited. All sleep studies will be interpreted manually by a sleep technician according to the standard criteria of the American Academy of Sleep Medicine Manual for the Scoring of Sleep and Associated Events and will be reviewed by certified physicians [11] . Patients will be evaluated against the inclusion and exclusion criteria, and their type of mobile phone and their previous experience with apps will be recorded. Information about the study will be provided to the participants, who will then have the opportunity to ask questions and have their doubts resolved. Patients accepted into the study will then sign the informed consent form.

### Initial Visit

During the initial visit, the patient will be evaluated by an ENT specialist, who will use oropharyngoscopy, rhinofibrolaryngoscopy, and the Marchesani protocol to examine the patient and to assess the Friedman stage and temporomandibular joint dysfunction. Patients with tonsil grade IV, complete nose obstruction, ankyloglossia, or temporomandibular joint dysfunction will be considered as selection failure.

### Second Visit

In the second visit, the patient will be given the same ENT examination as in the initial visit. Parameters of interest will be measured (weight, height, BMI, and neck and waist circumferences), and the patients will complete the questionnaires. The patients will then be randomized to the control group or intervention group. Patients in the intervention group will be instructed about the use of the app and exercises to perform.

The IOPI (model 2.1; IOPI Medical LLC, Carnation, WA) will be used to assess variables related to tongue function. The IOPI is a portable device that measures the amount of pressure exerted on a small air-filled bulb. The pressures obtained are expressed in kPa and displayed digitally on an LCD panel on the instrument. A series of LED lights representing percentages in 10% increments of a manually set pressure acts in combination with the built-in timer as a tool for measuring endurance. The IOPI has established high inter- and intrarater reliability, and has been used to measure tongue function by our group in an earlier publication [12]. To ensure accurate measurement, the calibration is checked and adjusted if needed before use for each participant. A new bulb will be used for every participant because of hygiene and to minimize measurement error because of possible compliance variations in the bulb after extended use.

The IOPI objectively measures maximum tongue and lip strength. Tongue strength is assessed by measuring the maximum pressure exerted when a person presses a disposable, standard-sized tongue bulb against the roof of the mouth. Lip strength is assessed by measuring the maximum pressure when the bulb is located between the cheek and closed teeth and the patient contracts the buccinator muscle without biting the bulb. Reference values have been obtained for a healthy population and are provided by the manufacturer [12].

Tongue strength is measured by obtaining maximal tongue elevation pressures. The patient is instructed to “place this bulb in your mouth on the midline of your tongue and push it against the roof of your mouth as hard as you can.” To maximize standard placement, the examiner demonstrates how to place the bulb along the central groove of the tongue blade. Previous research (12) indicates that maximal measures of tongue strength and endurance are best assessed with an unconstrained jaw, and participants will be encouraged to rest the incisors gently on the tubing of the IOPI bulb. Each test lasts 7-10 s, and all participants will be given verbal encouragement by the examiner for the entire test. The test will be performed 3 times by each participant, with a brief rest of about 30 s between each test while the examiner records the peak pressure obtained. The highest pressure across the 3 trials will be used as the maximal isometric pressure instead of the mean pressure, which is used by other researchers. Given the high correlation between the average and maximal pressure and that both are similarly related to oral-phase swallowing function, the use of the maximal pressure is more efficient in a clinical setting because it requires no calculation (10).

### Follow-up Visits

***Intervention group.*** Patients in the intervention group will perform exercises for about 20 min daily. The patients will attend follow-up visits after 1 month (visit 3) and 2 months later (visit 4). During these visits, the parameters of interest will be measured, and participants will complete the questionnaires and be asked about their performance of the exercises.

***Control group.*** Patients in the control group will attend follow-up visits after 1 month (visit 3) and 2 months later (visit 4). During these visits, the parameters of interest will be measured, and participants will complete the questionnaires and confirm that no other therapies are being followed.

The final visit will be at 3 months in both groups. During this visit, the parameters of interest will be measured and laboratory polysomnography or poligraphy will be performed on patients, and all participants will complete the questionnaires.

The total study duration for each patient will be 3 months.

## Discontinuation Criteria

Patients will be excluded or discontinued from the study if they:

- do not perform ≥ 85% of the sessions (intervention group),
- are lost to follow-up for not attending hospital (both groups),
- lose ≥ 5% of body weight during the 3 months of the study (both groups).

## Statistical Analysis

Data will be collected in a database. Nominal variables will be described by their frequency distribution. Quantitative variables will be assessed by calculating the arithmetic mean and standard deviation. Baseline characteristics will be compared between groups using 2-tailed paired *t* tests for continuous variables and the χ^2^ or Fisher exact test for nominal variables. For variables with a skewed distribution, the Mann–Whitney *U* test will be used. Two-way repeated-measures analysis of variance and the Tukey test will be used to compare differences within and between groups in variables measured at the baseline and after 3 months. Pearson correlational analysis will be used to identify associations between changes in the AHI and changes in possible explanatory variables, including BMI, and waist and neck circumferences. A *P* value of 0.05 will be considered significant. IBM SPSS Statistics for Windows software (version 20; IBM Corp., Armonk, NY, USA) will be used for statistical analysis.

The main parameters of interest will include polysomnography and polygraph findings at the beginning and end of the study, IOPI scores at the beginning and the end of the study, Epworth and Pittsburgh questionnaire scores at all visits, body weight (kg), height (cm), BMI (kg/m^2^), and neck and waist circumferences (cm) at all visits.

## Ethical Approval

After evaluation by the Investigation Ethical Committee Costa del Sol and our revision of the protocol, the study protocol was approved with the code AWGAPN-2019-01. Before starting the trial, all participants will be required to sign and date all consent forms, which have been approved by the ethical review committee.

### Access to Data and Data Protection

To guarantee data confidentiality, the original data will be stored within each hospital and only the investigators and the Investigation Ethical Committee will have access to the data. This study is based on the ethical principles of the last revision of the Helsinki Declaration and Good Clinical Practice. All participants will be ensured of the confidentiality of all data (Spanish Organic Law Personal Data Protection 15/1999), and all information will be used only for the aims specified in the trial. Personal data that could identify patients will be separated from the rest of the information obtained in the trial. Every patient will have an identification number, which will be used instead of the name in the database.

# Utility

This app may be helpful for improving the AHI and quality of life, and for reducing clinical symptoms and the need for treatment in patients with OSAHS.

## Schedule

| **Activity (months)** | **1** | **2** | **3** | **4** | **5** | **6** | **7** | **8** | **9** | **10** | **11** | **12** |
| --- | --- | --- | --- | --- | --- | --- | --- | --- | --- | --- | --- | --- |
| Starting, database design, and coordination |  |  |  |  |  |  |  |  |  |  |  |  |
| Recruitment |  |  |  |  |  |  |  |  |  |  |  |  |
| Follow-up |  |  |  |  |  |  |  |  |  |  |  |  |
| Data collection and entry into the database |  |  |  |  |  |  |  |  |  |  |  |  |
| Statistical analysis and reporting results |  |  |  |  |  |  |  |  |  |  |  |  |

## References

1. Mendes F, Marone S, Duarte B, Arenas A. Epidemiologic profile of patients with snoring and obstructive sleep apnea in a university hospital. Int Arch Otorhinolaryngol 2014 Apr;18(2):142-145. [PMID: 25992080]
2. Camacho M, Certal V, Abdullatif J, Zaghi S, Ruoff CM, Capasso R, Kushida CA. Myofunctional therapy to treat obstructive sleep apnea: a systematic review and meta-analysis. Sleep 2015 May 1;38(5):669-675. [PMID: 25348130]
3. Korhan I, Gode S, Midilli R, Basoglu OK. The influence of the lateral pharyngeal wall anatomy on snoring and sleep apnoea. J Pak Med Assoc 2015 Feb;65(2):125-130. [PMID: 25842544]
4. O’Connor Reina C, Plaza G, Ignacio-Garcia JM, Jardin PB, Garcia-Iriarte MT, Casado-Morente JC, Gonzalez ED, Rodriguez-Reina A. New mHealth application software based on myofunctional therapy applied to sleep-disordered breathing in non-compliant subjects. Sleep Sci Pract 2020 Feb 5;4:3.
5. Isetta V, Torres M, González K, Ruiz C, Dalmases M, Embid C, Navajas D, Farré R, Montserrat JM. A new mHealth application to support treatment of sleep apnoea patients. J Telemed Telecare 2017 Jan;23(1):14-18. [PMID: 26672606]
6. Camacho M, Robertson M, Abdullatif J, Certal V, Kram YA, Ruoff CM, Brietzke SE, Capasso R. Smartphone apps for snoring. J Laryngol Otol 2015 Oct;129(10):974-979. [PMID: 26333720]
7. Iftikhar IH, Bittencourt L, Youngstedt SD, Ayas N, Cistulli P, Schwab R, Durkin MW, Magalang UJ. Comparative efficacy of CPAP, MADs, exercise-training, and dietary weight loss for sleep apnea: a network meta-analysis. Sleep Med 2017 Feb;30:7-14. [PMID: 28215266]
8. Guimaraes KC. Orofacial myofunctional treatment. In: Pulso, editor. Apneia e Ronco; 2009. 53-63.
9. Marchesan I. Lingual frenulum protocol. Int J Orofac Myol 2012 Nov;38:89-103. [PMID: 23367525]
10. Guimarães KC, Drager LF, Genta PR, Marcondes BF, Lorenzi-Filhoy G. Effects of oropharyngeal exercises on patients with moderate obstructive sleep apnea syndrome. Am J Respir Crit Care Med 2009 May 15;179(10):962-966. [PMID: 19234106]
11. Berry RB, Gamaldo CE, Harding SM, et al. AASM Scoring Manual Version 2.2 Updates: New Chapters for Scoring Infant Sleep Staging and Home Sleep Apnea Testing. *J Clin Sleep Med*. 2015;11(11):1253-1254. [PMID: 26446251]
12. O’Connor-Reina C, Plaza G, Garcia-Iriarte MT, Ignacio-Garcia JM, Baptista P, Casado-Morente JC, De Vicente E. Tongue peak pressure: a tool to aid in the identification of obstruction sites in patients with obstructive sleep apnea/hypopnea syndrome. Sleep Breath 2019 Mar;24(1):281-286. [PMID: 31745755]

## Multimedia appendix 2

Screenshots from the app

# EXERCISES AND VIDEOS

For reasons of hygiene, we recommend that the patient covers the smartphone screen with cling film or a cleaning wipe for all exercises.

## Multimedia appendix 3

## Exercise 1. Chromatic Snake

With your elbows touching your body, hold the phone with both hands as you would when holding a mirror in front of your face.

Follow the order of the lights and music. Stick your tongue out and press the screen with it following the pattern of the lights.

Perform the exercise 15 times.

VIDEO 1

## Multimedia appendix 4

## Exercise 2. Snake

With your elbows touching your body, hold the phone with both hands as you would when holding a mirror in front of your face.

Stick your tongue out and press the screen with it for 5 s and release. The tongue should not touch your teeth or lips.

Perform the exercise 15 times.

VIDEO 2

## Multimedia appendix 5

## Exercise 3. Chameleon Up

Place the phone 10 cm in front of you, as if it were a mirror. Stick out your tongue and press on the square at the bottom. The tongue must not touch your teeth or lips.

Place your tongue upwards and keep the pressure on the screen for 5 s, release your tongue and then close your mouth and rest.

Perform the exercise 15 times.

VIDEO 3

## Multimedia appendix 6

## Exercise 4. Chameleon Down

Place the phone at a certain distance in front of you, as if it were a mirror. Stick out your tongue and press on the square at the bottom. The tongue must not touch your teeth or lips.

Place your tongue down and keep pressing the screen for 5 s, release your tongue and then close your mouth and rest.

Perform the exercise 15 times.

VIDEO 4

## Multimedia appendix 7

## Exercise 5. Tongue Left Cheek

Press the inside of the left cheek with the tip of your tongue. Allow the screen of your phone to touch your cheek gently.

Contract the muscles of your cheek while pushing with the tongue toward the cheek. Keep the pressure for 5 s.

Perform the exercise 15 times.

VIDEO 5

## Multimedia appendix 8

## Exercise 6. Tongue Right Cheek

Press the inside of the right cheek with the tip of your tongue. Allow the screen of your phone to touch your cheek gently.

Contract the muscles of your cheek while pushing with the tongue toward the cheek. Keep the pressure for 5 s.

Perform the exercise 15 times.

VIDEO 6

## Multimedia appendix 9

## Exercise 7. Pressure Under Chin

Hold the phone with both hands with the screen facing up. If you have a beard, place a finger between your phone and your jaw.

Sitting upright place the phone under your chin so there is no contact with the chin.

Bend your head forward like you are going to drink from a cup and contact your chin on the phone.

Keep the contact and move your head from side to side during 10 s with your mouth closed. Lift your head and rest. Repeat the exercise 15 times.

VIDEO 7

## Multimedia appendix 10

## Exercise 8. Left Mandibular Pressure

Bring the phone closer to the angle of the jaw on the left side, as if you were talking on the phone but with the screen to the outside.

Turn your jaw toward the phone and keep the pressure with your index finger on the screen for 5 s. Say “I” while doing this. Release the pressure and rest.

Perform the process 15 times.

VIDEO 8

## Multimedia appendix 11

## Exercise 9. Right Mandibular Pressure

Bring the phone closer to the angle of the jaw on the right side, as if you were talking on the phone but with the screen to the outside.

Turn your jaw toward the phone and keep the pressure with your index finger on the screen for 5 s. Say “I” while doing this.

Perform the process 15 times.

VIDEO 9


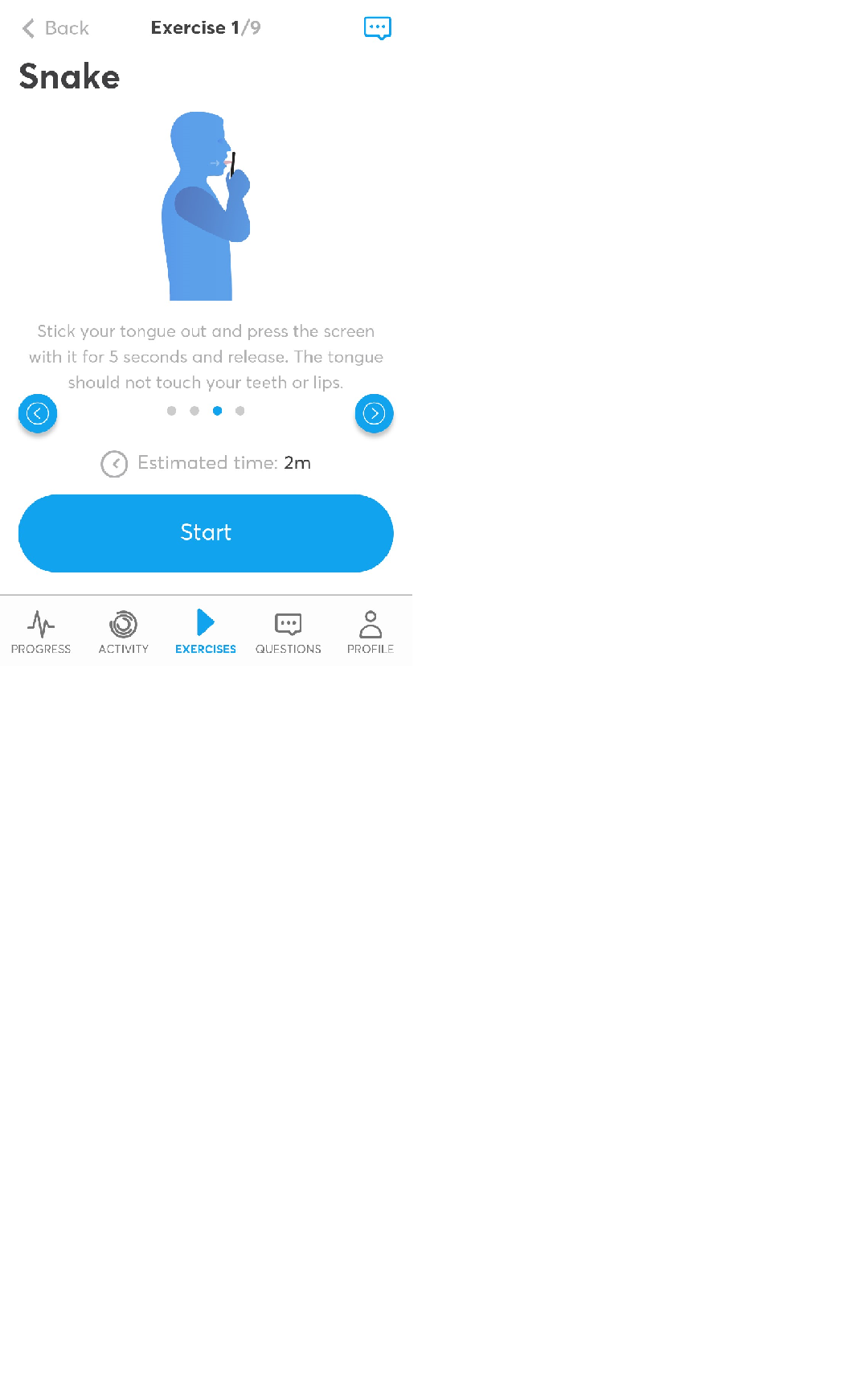


Figure 5


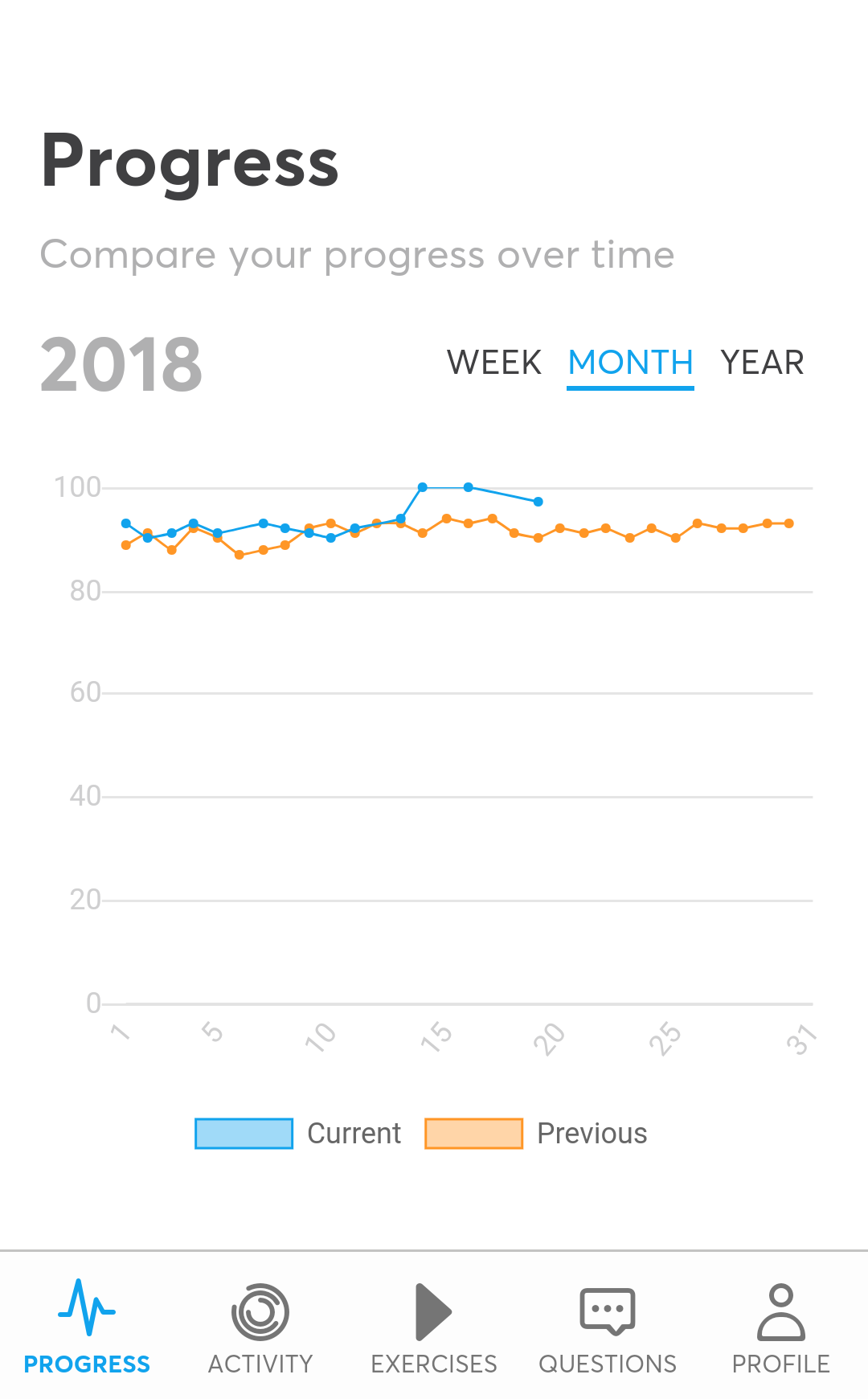


Figure 6


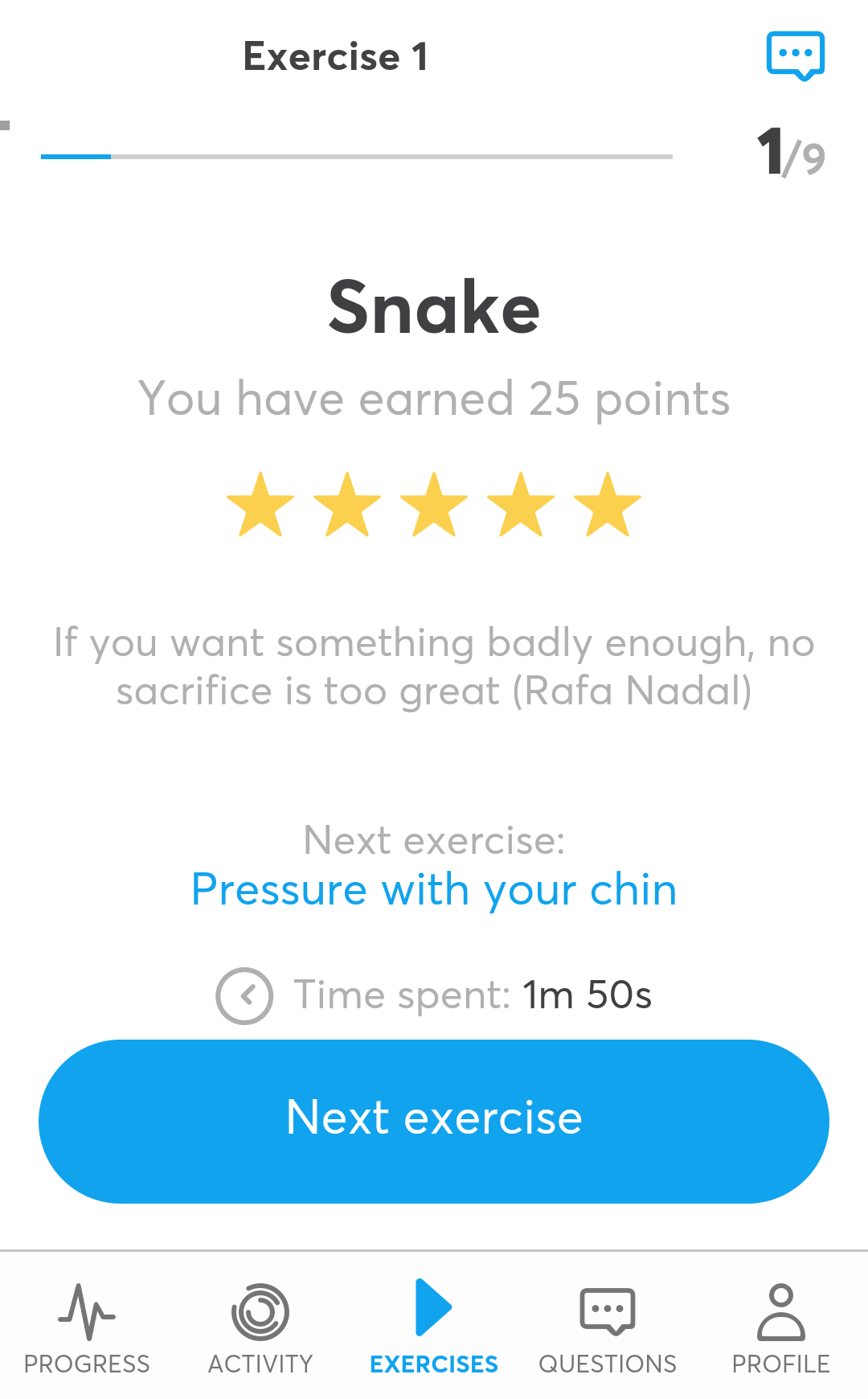


Figure 7


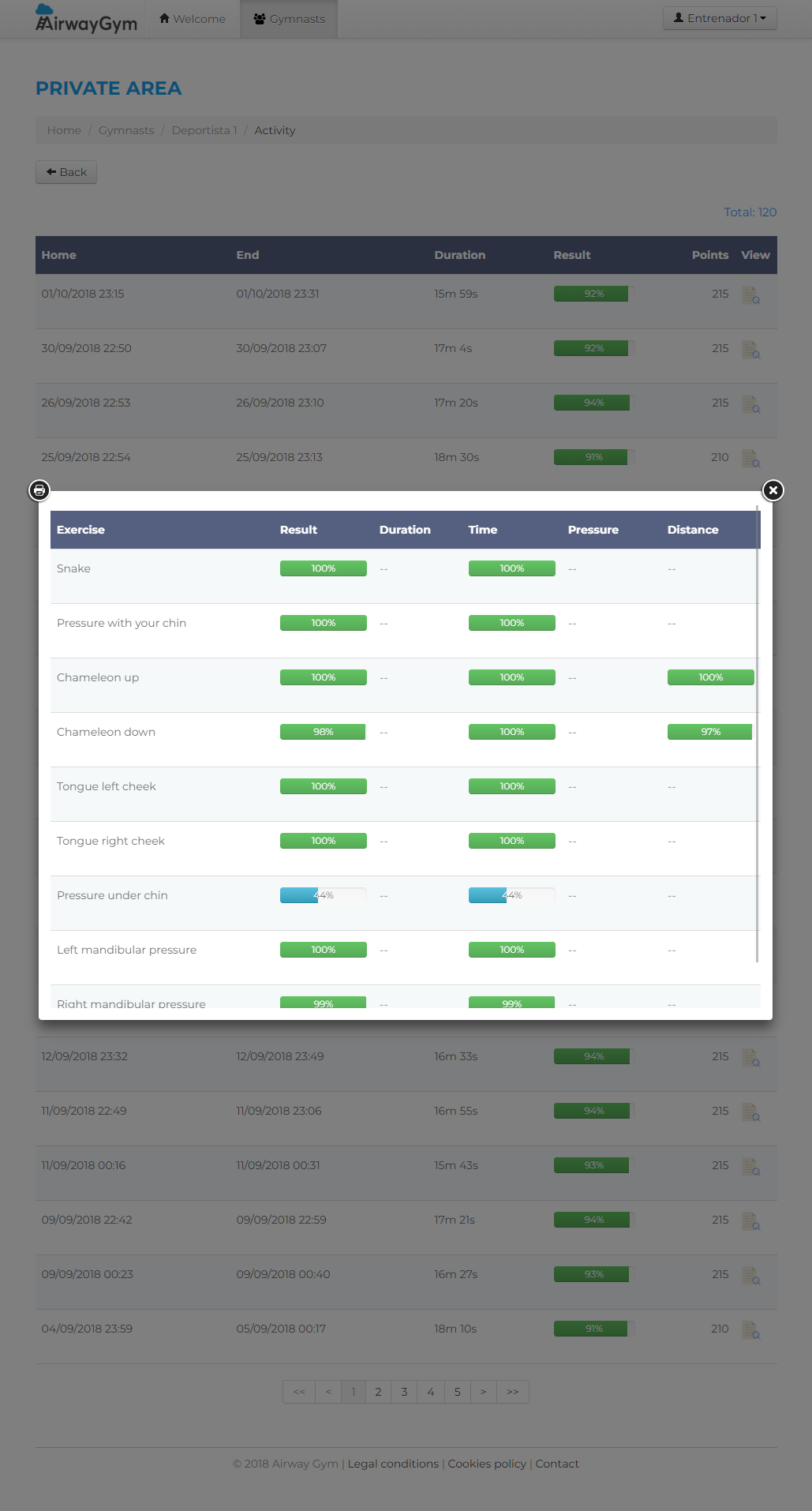


Figure 8
