# Supplementary figures and images for: Myofunctional Therapy App for Severe Apnea–Hypopnea Sleep Obstructive Syndrome: Pilot Randomized Controlled Trial
Source: JMIR Mhealth Uhealth. 2020 Nov 9;8(11):e23123. doi: 10.2196/23123 (PMC7683258; doi:10.2196/23123)

## Slide 1
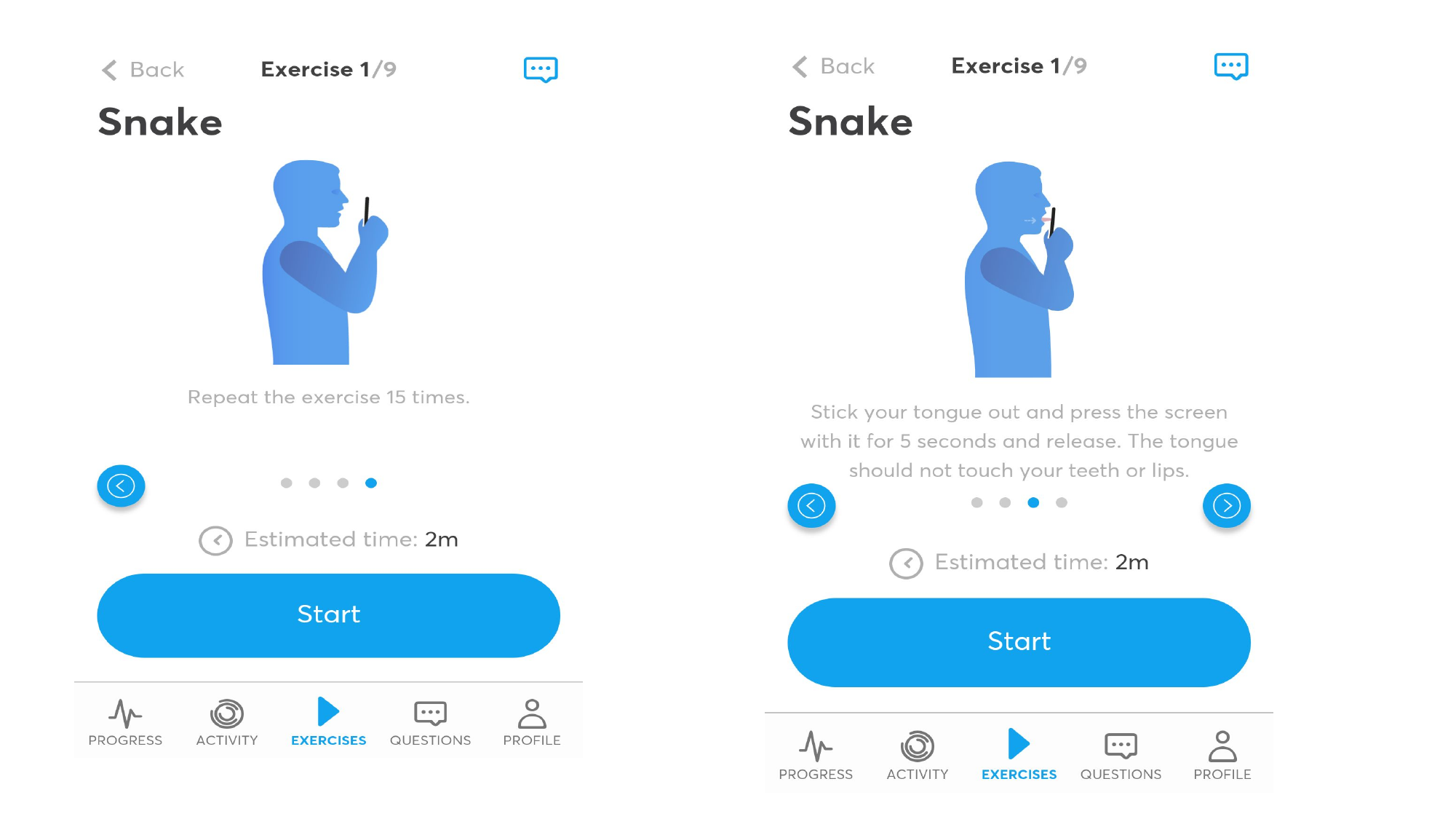

#

## Slide 2
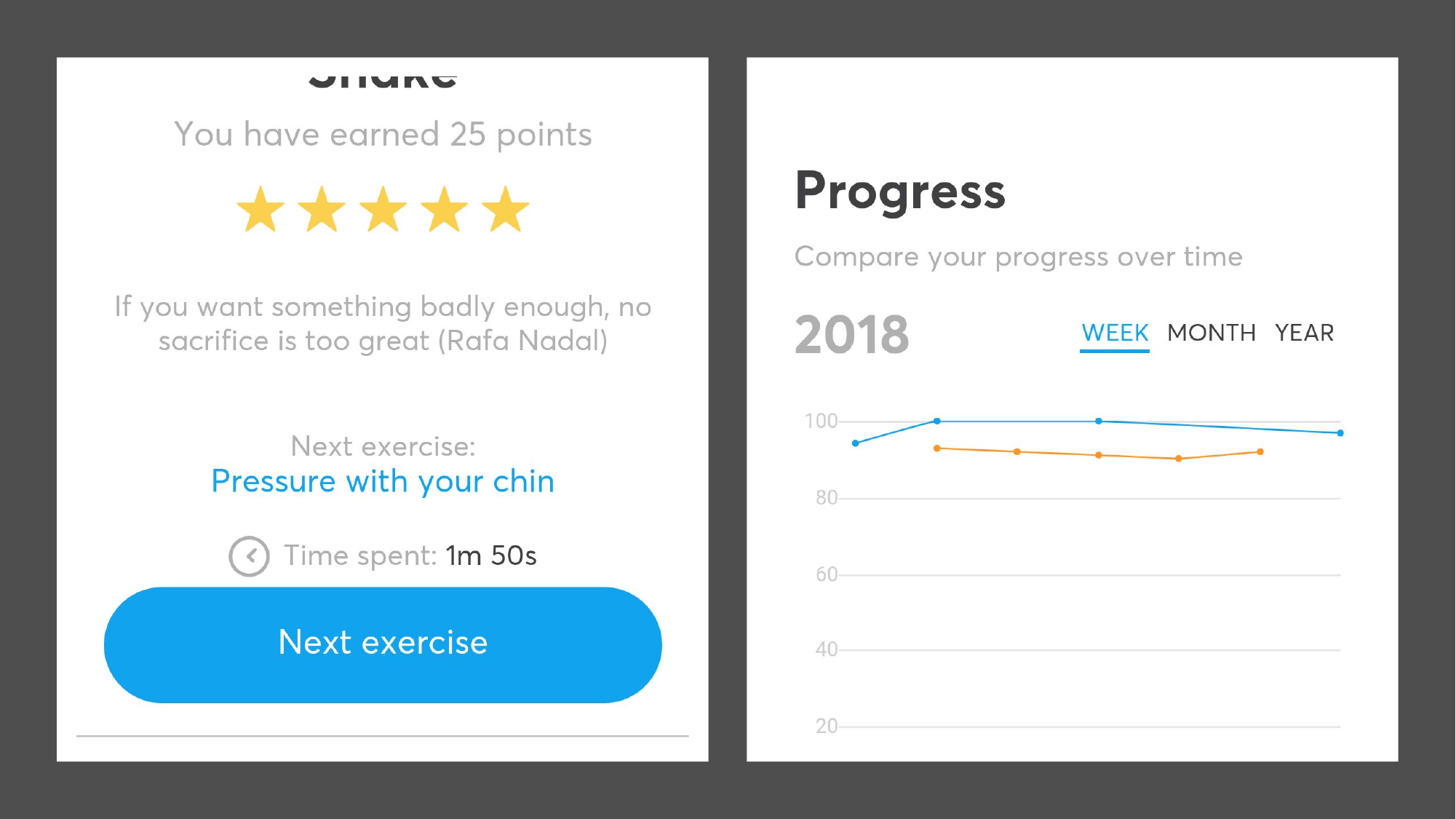

## Slide 3
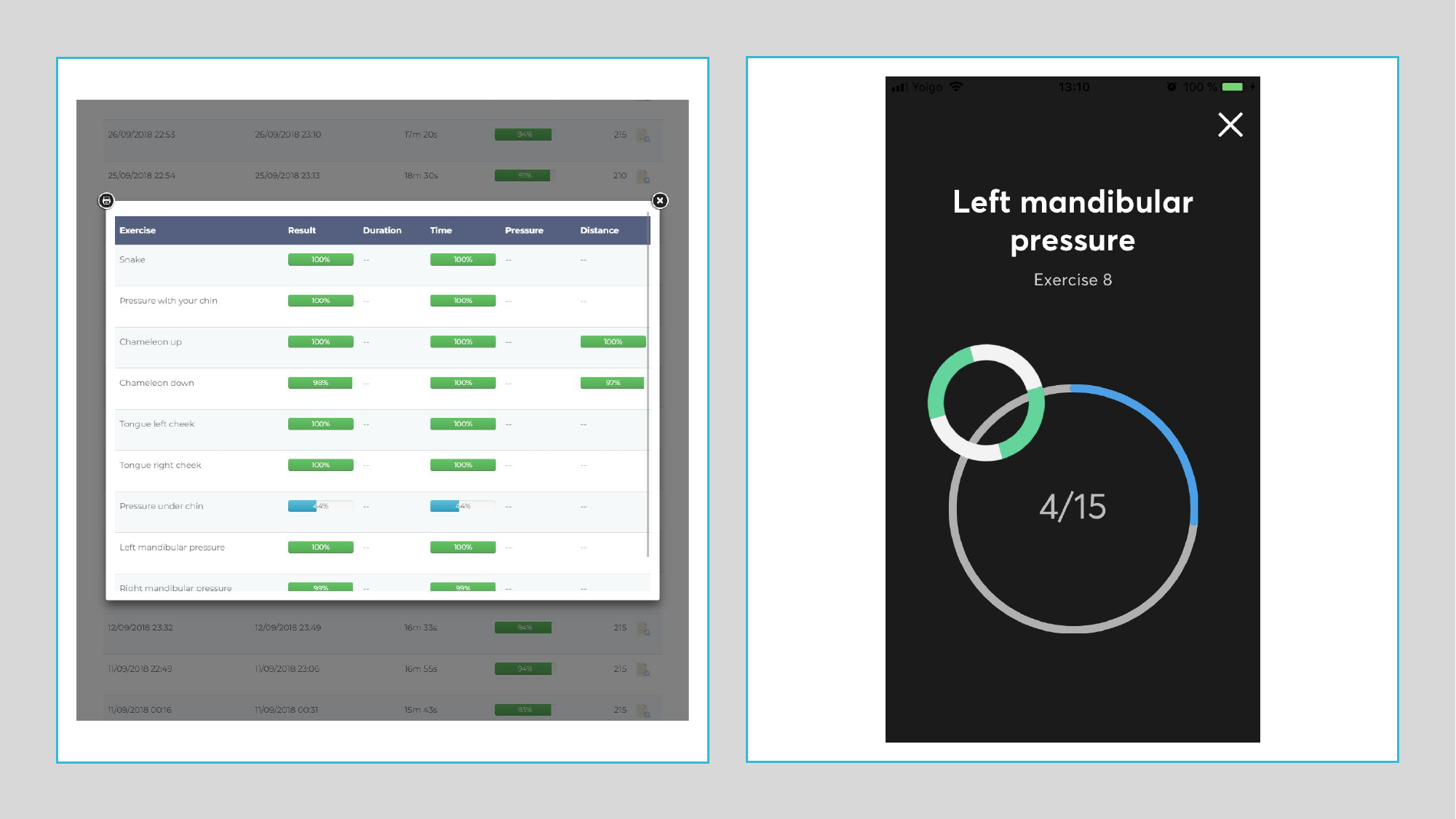

Supplement: Multimedia Appendix 3 [file mhealth_v8i11e23123_app3.pptx]
